# Are we any closer to optimising amblyopia treatment?

**DOI:** 10.1002/ctm2.70080

**Published:** 2024-12-01

**Authors:** Frank Antony Proudlock, Irene Gottlob

**Affiliations:** ^1^ Ulverscroft Eye Unit School of Psychology and Vision Sciences University of Leicester Robert Kilpatrick Clinical Sciences Building Leicester Royal Infirmary Leicester UK; ^2^ Department of Neurology Cooper University Health Care Cooper Medical School of Rowan University Camden New Jersey USA

**Keywords:** amblyopia, anisometropia, extended optical treatment, glasses‐wear, patching, refractive adaptation, strabismus

## 1 COMMENTARY

The visual cortex is normally organised so that projections from corresponding retinal locations in both eyes, representing the same visual space, are adjacent in the brain. This arrangement enables direct comparison of information from each eye, helping to identify retinal disparities caused by the subtle differences in perspective between the two eyes, which is crucial for generating our three‐dimensional perception of the world. This precise arrangement is easily disturbed if unequal inputs into the eyes occur during the period of high brain plasticity in the early years of life.

Two common issues—strabismus (eye misalignment) and anisometropia (unequal refractive power between the eyes)—can lead to the brain actively suppressing input from one eye. This phenomenon, called unilateral amblyopia, is the most common visual disorder in children, with an estimated global prevalence of 99.2 million in 2019.^1^ This number is expected to rise to 221.9 million by 2040^1^ making amblyopia a major public health concern contributing significantly to healthcare costs in paediatric eye care.

The standard treatments for amblyopia are wearing corrective glasses, followed by patching the better‐seeing eye.^2^ However, patching treatment can cause discomfort, temporary vision reduction, and social stigma, leading to poor adherence among children.^2^ To address these challenges, researchers have explored alternative treatments. One approach, extended optical treatment (EOT), involves prolonging glasses wear to improve vision before patching starts. Other less common alternatives include administering atropine eye drops to blur vision in the stronger eye or using computer‐based dichoptic activities.^2^

A 2020 meta‐analysis reviewed 23 clinical trials comparing multiple amblyopia treatments,^3^ which form the basis for current treatment guidelines.^4^, ^5^ Despite the range of treatments available, success remains limited, with around 30% of children treated clinically failing to achieve an amblyopic eye best‐corrected visual acuity (BCVA) of 6/12 even after years of patching.^6^ Additionally, there is a need for more personalised treatment strategies tailored to a child's age, type and severity of amblyopia, and social background. Here we discuss current knowledge about best approaches towards the mainstay treatments of glasses and patch wear.

## 2 GLASSES WEARING

EOT seeks to optimise visual inputs to both eyes, gradually improving vision, before transitioning to patching. A meta‐analysis of 20 studies demonstrates that EOT improves vision by on average 2−3 logMAR lines.^7^ Consequently, EOT is widely recommended in clinical guidelines.^4^, ^5^ Until recently, however, no studies have directly compared amblyopia treatments with and without EOT.

**FIGURE 1:**
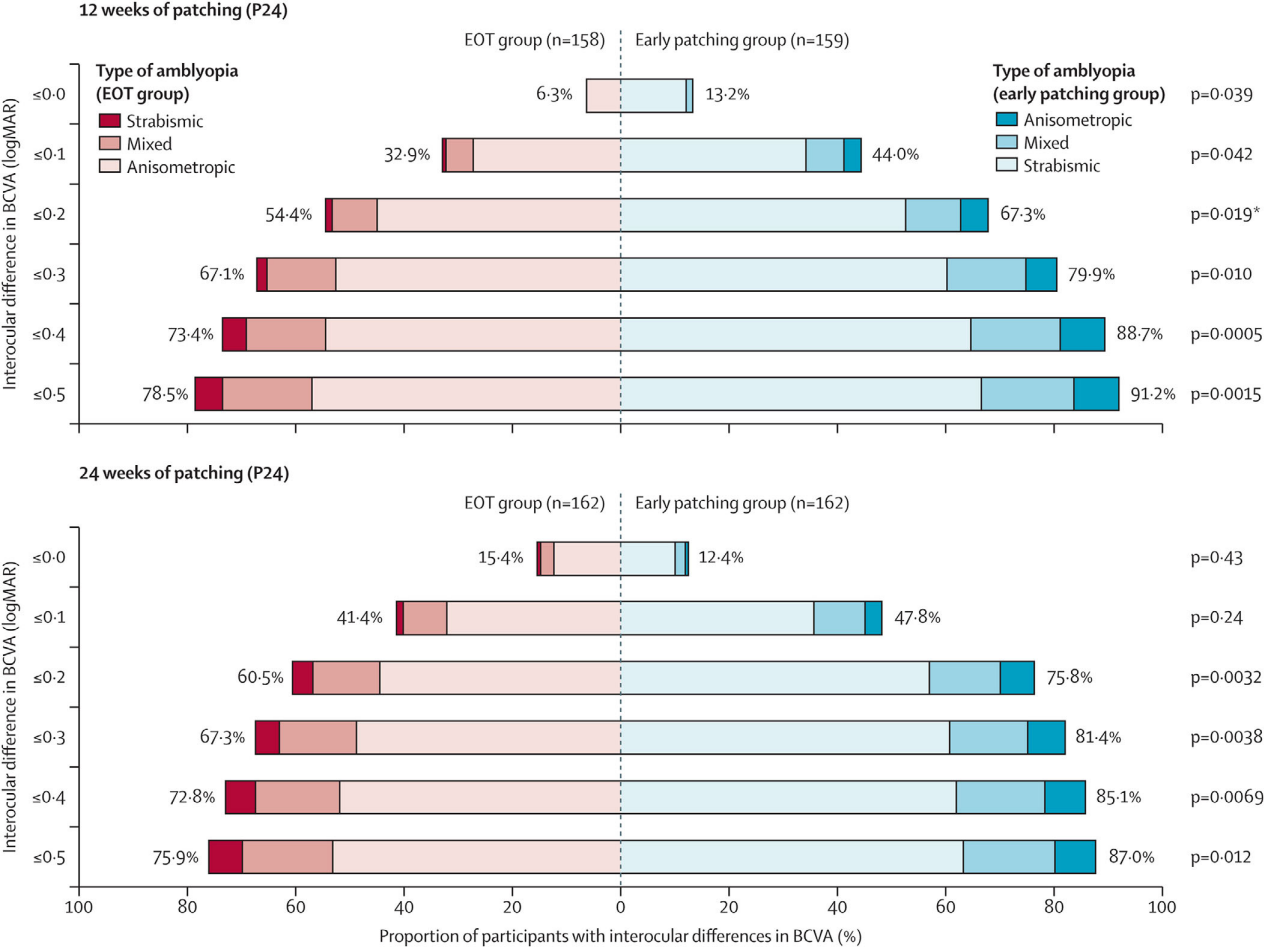
Proportion of participants reaching interocular differences in visual acuity at the primary outcome and end of the EuPatch^8^ study. Different shades of colour show the breakdown by type of amblyopia (anisometropic, mixed, strabismic) for the primary outcome (12 weeks of patching) and the final outcome (24 weeks of patching). Statistical comparisons between the extended optical treatment (EOT) group and the early patching group were done with *χ*2 tests. For each panel, the proportions of participants reaching the thresholds of improvement indicated on the y‐axis are provided for the EOT group (red) and for the early patching group (blue). The percentage values provided are for all participants in each group. *EOT, extended optical treatment; log*MAR*, logarithm of the minimum angle of resolution. The primary outcome (i.e. ≤0.20 log*MAR* interocular difference at 12 weeks). BCVA, best corrected visual acuity. The figure was published in Proudlock et al. 'Extended optical treatment versus early patching with an intensive patching regimen in children with amblyopia in Europe (EuPatch): a multicentre, randomised controlled trial', Lancet. 2024 Jun 22;403(10445):2694, Copyright Elsevier (2024).

In our recent clinical trial, the EuPatch study, 334 children from 30 hospitals across Europe, were randomly assigned to two groups: one receiving EOT and the other starting patching earlier.^8^ The earlier patching (EP) group, as a whole, achieved significantly better outcomes, with higher success rates after both 12‐ and 24‐weeks of patching (Figure 1). Parents of children in the EP group also reported a more positive attitude toward patching, likely due to the faster visual improvement during the patching phase.

During the EOT phase, before patching commenced, the 163 children in the EOT group exhibited varying degrees of visual improvement, mostly improving early on. During 18 weeks of EOT, 64% of improvement occurred within 6 weeks and 88% within 12 weeks.

**FIGURE 2:**
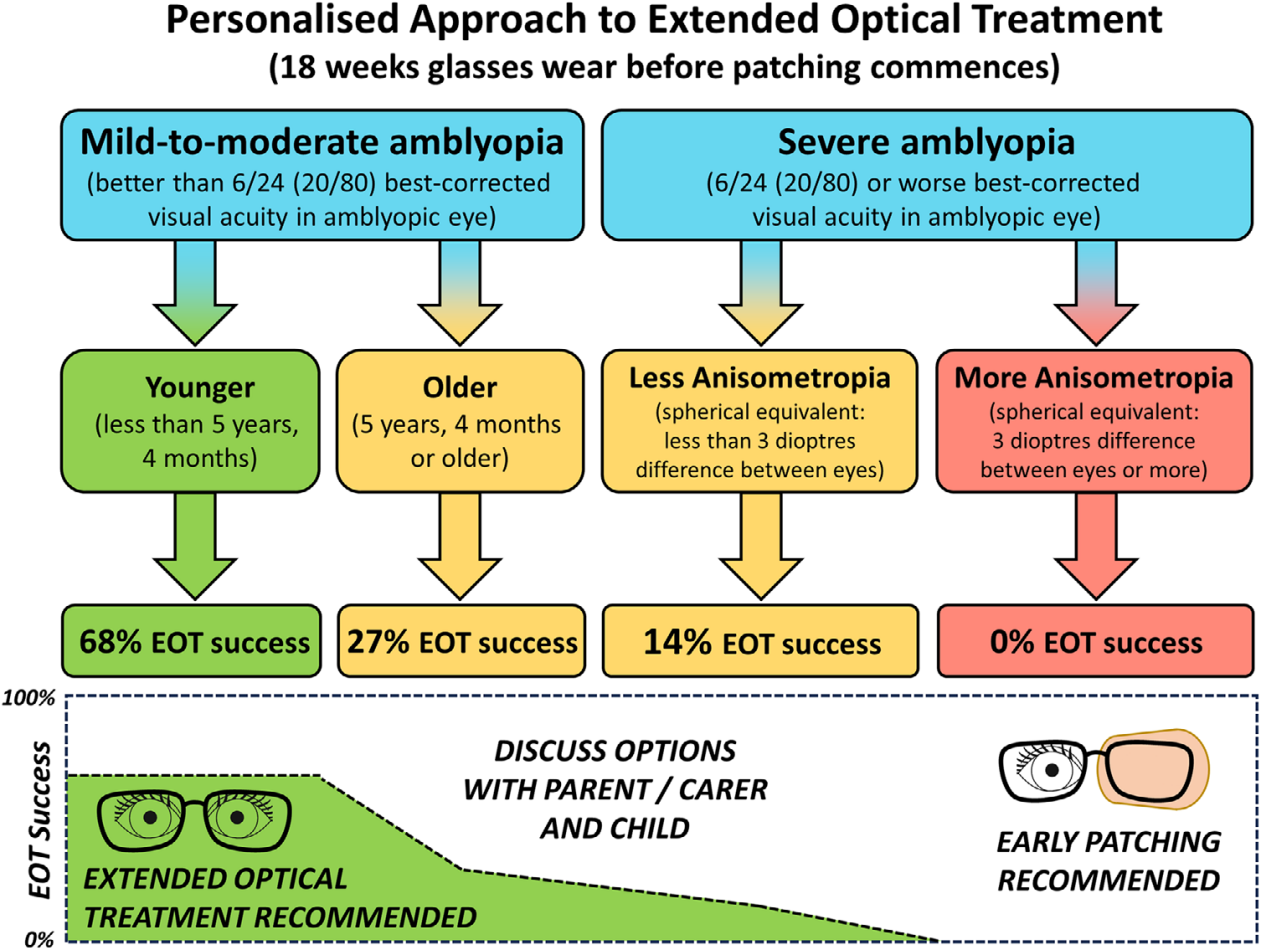
Information to aid clinical decision‐making in the personalised use of extended optical treatment at the start of treatment. Using recursive partitioning, we identified the optimal split of predictor variables that would partition the data into outcome groups. BCVA, best‐corrected visual acuity; EOT, extended optical treatment.

We were able to develop a personalised approach to EOT, tailored to the child's individual characteristics (Figure 2). EOT is recommended for younger children (under 5 years, 4 months) with mild‐to‐moderate amblyopia (amblyopic eye BCVA < 0.6 logMAR), who demonstrated a 68% success rate for EOT. For children with severe amblyopia (BCVA ≥0.6 logMAR) and significant anisometropia (spherical equivalent difference between the eyes: ≥3 diopters), however, early patching after a brief period of glasses wear (3 weeks) is advised, since the success rate was 0%. In the remaining children, the success of EOT was low, at 27% in older mild‐to‐moderate amblyopes, and 14% in severe amblyopes with less anisometropia. In these children, the likelihood of success with EOT versus early patching should be discussed with families and adjusted to individual needs.

This information marks a significant advancement towards achieving a personalised approach to treating amblyopia with glasses and offers valuable guidance for clinicians and parents in selecting the most appropriate treatment strategy.

## 3 PATCHING

Clinical guidelines, such as those from the American Academy of Ophthalmology,^4^ recommend low patching doses of 2 h per day for mild‐to‐moderate amblyopia and 6 h per day for severe amblyopia. However, real‐world outcomes for 589 children prescribed these regimens were poorer than in trials.^9^, ^10^ For mild‐to‐moderate amblyopes prescribed 2 h of patching daily for 32 weeks, the success rate was 71% in a clinical setting compared to 81% in trials.^9^, ^10^ For severe amblyopes prescribed 6 h daily, success rates were 40% in clinical settings and 67% in trials.^9^, ^10^ In our cohort, after 24 weeks of patching, the success rate was 82% for the EP group and 72% for the EOT group. Notably, 64% of the cohort included severe amblyopes, although severity was based on pre‐glasses criteria.

Possible reasons for the superior outcomes we observed, after only 24 weeks of patching, could be the higher patching dose we prescribed of 10 h per day, 6 days a week supplemented with educational and motivational materials to improve adherence.

Our findings suggest that commonly used lower patching regimens may be suboptimal and that higher patching doses may have been dismissed prematurely as a valid treatment strategy. The rapid visual improvements seen with early initiation of patching, combined with educational and motivational support, could make higher doses more effective and less burdensome for parents. Furthermore, quicker gains in visual acuity are likely to enhance motivation and adherence to treatment.

## 4 CONCLUSION

Our results indicate that while EOT may not be beneficial for every child, there is a solid evidence base for a personalised approach to its use, especially for young children with mild‐to‐moderate amblyopia.

A more granular analysis of personal factors such as age, BCVA, refraction, type of amblyopia, stereovision, and socioeconomic background, could be helpful in identifying the best treatment modality, including patching dosage, ideal frequency of clinical visits, and the most appropriate motivational/educational interventions tailored to each child.
